# Extracellular CIRP dysregulates macrophage bacterial phagocytosis in sepsis

**DOI:** 10.1038/s41423-022-00961-3

**Published:** 2022-12-05

**Authors:** Mian Zhou, Monowar Aziz, Hao-Ting Yen, Gaifeng Ma, Atsushi Murao, Ping Wang

**Affiliations:** 1 Center for Immunology and Inflammation, The Feinstein Institutes for Medical Research, Manhasset, NY USA; 2 Departments of Surgery and Molecular Medicine, Zucker School of Medicine at Hofstra/Northwell, Manhasset, NY USA

**Keywords:** βPIX, eCIRP, Macrophage, Phagocytosis, Rac1, STAT3, Immunology, Bacterial infection

## Abstract

In sepsis, macrophage bacterial phagocytosis is impaired, but the mechanism is not well elucidated. Extracellular cold-inducible RNA-binding protein (eCIRP) is a damage-associated molecular pattern that causes inflammation. However, whether eCIRP regulates macrophage bacterial phagocytosis is unknown. Here, we reported that the bacterial loads in the blood and peritoneal fluid were decreased in CIRP^−/−^ mice and anti-eCIRP Ab-treated mice after sepsis. Increased eCIRP levels were correlated with decreased bacterial clearance in septic mice. CIRP^−/−^ mice showed a marked increase in survival after sepsis. Recombinant murine CIRP (rmCIRP) significantly decreased the phagocytosis of bacteria by macrophages in vivo and in vitro. rmCIRP decreased the protein expression of actin-binding proteins, ARP2, and p-cofilin in macrophages. rmCIRP significantly downregulated the protein expression of βPIX, a Rac1 activator. We further demonstrated that STAT3 and βPIX formed a complex following rmCIRP treatment, preventing βPIX from activating Rac1. We also found that eCIRP-induced STAT3 phosphorylation was required for eCIRP’s action in actin remodeling. Inhibition of STAT3 phosphorylation prevented the formation of the STAT3-βPIX complex, restoring ARP2 and p-cofilin expression and membrane protrusion in rmCIRP-treated macrophages. The STAT3 inhibitor stattic rescued the macrophage phagocytic dysfunction induced by rmCIRP. Thus, we identified a novel mechanism of macrophage phagocytic dysfunction caused by eCIRP, which provides a new therapeutic target to ameliorate sepsis.

## Introduction

Sepsis is the leading cause of death among patients in the intensive care unit [1]. A broad range of cytokines, chemokines, and effector molecules are released from cells in response to pathogen-associated molecular patterns (PAMPs) and damage-associated molecular patterns (DAMPs) during the initial phase of sepsis to exacerbate inflammation and tissue injury [2, 3]. The immunosuppressive phase of sepsis is characterized by reduced activity and numbers of phagocytic cells and increased numbers of immunoregulatory and immunotolerant cells [4, 5]. Phagocytosis is a crucial cellular mechanism for elimination of pathogens and infected or damaged cells, promoting tissue regeneration and inflammation resolution and restoring homeostasis [6]. Immunosuppressed patients have difficulties eradicating invading pathogens due to impaired phagocytosis of bacteria, which renders them susceptible to life-threatening nosocomial infection [7, 8].

Macrophages play an essential role in the phagocytosis of bacteria [6]. Macrophages express receptors that recognize microbes. These receptors are categorized as opsonic and nonopsonic [6]. Opsonic receptors, mainly Fc receptors (FcRs), bind antibodies coating the pathogen surface. Nonopsonic receptors directly recognize PAMPs or act as scavenger receptors, such as scavenger receptor-A, macrophage receptor with collagenous structure (MARCO), scavenger receptor with C-type lectin, and CD36 [6]. Once a microbe binds to receptors on a phagocyte, the plasma membrane of the phagocyte in the region of the receptors begins to redistribute. It extends a cup-like structure around the microbe, followed by formation of a vesicle called a phagosome [6]. The phagosome later fuses with a lysosome for complete cargo digestion [6]. Phagocytosis requires energetically demanding actin filament polymerization and depolymerization processes that facilitate phagocytic receptor mobility, pathogen detection, and engulfment. Actin remodeling is mediated by dynamic changes in the activity of Rho GTPases (RhoA, Rac1, and Cdc42) [6, 9]. Both FcR- and scavenger receptor-mediated phagocytosis induce profound actin-dependent membrane remodeling. Actin remodeling participates in target internalization and phagosome formation in the cytoplasm.

Cold-inducible RNA-binding protein (CIRP) is an 18-kDa nuclear protein expressed in various types of cells that acts as an RNA chaperone protein [10]. CIRP mRNA and protein expression in sepsis is significantly increased in the liver and lungs [11, 12], which leads to increased extracellular CIRP (eCIRP) levels in the circulation. Macrophages were identified as the primary source of eCIRP following endotoxemia or hypoxia [11]. eCIRP is released via a passive release pathway through secondary necrosis and an active release pathway through lysosomal exocytosis and exosomes [11, 13]. eCIRP levels are increased in the serum of mice with sepsis, hemorrhagic shock, or ischemia‒reperfusion injury [10, 11]. eCIRP induces macrophages to release proinflammatory cytokines and chemokines via Toll-like receptor 4 (TLR4) and triggering receptor expressed on myeloid cells-1 (TREM-1) pathways [11, 14]. In addition to the proinflammatory role of eCIRP, its immunosuppressive function was recently reported. We demonstrated that eCIRP released during a late stage of sepsis promoted endotoxin tolerance in macrophages by recognizing interleukin-6 receptor (IL-6R) and activating the STAT3 pathway [15]. Given the impact of eCIRP on inducing immune tolerance in macrophages, elucidation of its role in the phagocytic function of macrophages in sepsis is of scientific and translational significance. Here, we identified a novel eCIRP-mediated STAT3-βPIX-Rac1 signaling pathway involved in architectural changes in macrophages that led to impaired bacterial clearance in sepsis. Our findings address the complex pathophysiology of sepsis and identify a novel therapeutic avenue by targeting eCIRP to protect against sepsis.

## Materials and methods

### Reagents

pHrodo *E. coli* (K12 strain) bioparticle conjugates (catalog no: P35366 for green florescence, and P35361 for red florescence) and an opsonizing reagent (catalog no: E2870) were purchased from Thermo Fisher Scientific (Carlsbad, CA). Cell culture media were purchased from Thermo Fisher Scientific. The antibodies for Western blotting included anti-ARP2 (catalog no: 5614), anti-p-cofilin (catalog no: 3313), anti-total cofilin (catalog no: 5175), anti-βPIX (catalog no: 4515), anti-mouse p-STAT3 (Tyr705, catalog no: 9131), and anti-total STAT3 (catalog no: 9139); these were obtained from Cell Signaling Technologies (Danvers, MA). Anti-β-actin antibodies (clone AC-15, catalog no: A5441), an anti-Rac1 magnetic bead conjugate (catalog no: 16-319), and anti-Rac1 antibodies (catalog no: 05-389) were purchased from Millipore-Sigma (St. Louis, MO). A Rac1 activation magnetic bead pulldown assay kit (catalog no: 17-10394) was purchased from Millipore-Sigma (St. Louis, MO). Infrared dye-labeled secondary antibodies were obtained from Li-Cor Biosciences (Lincoln, NE). The STAT3 inhibitor stattic (catalog no: sc-202818) was purchased from Santa Cruz Biotechnology (Santa Cruz, CA). The Rac1 inhibitor NSC 23766 (catalog no: 13196) was purchased from Cayman Chemicals (Ann Arbor, MI). A MemBrite cell surface staining kit was purchased from Biotium (catalog no: 30093-T, Fremont, CA).

### Experimental animals and sepsis induction

Male C57BL/6 mice were purchased from Charles River Laboratories (Fairfield, NJ). CIRP^−/−^ mice on the C57BL/6 background were provided by Dr. Jun Fujita (Kyoto University, Kyoto, Japan) as a kind gift [16]. Age-matched, healthy male mice (9–12 weeks old) were used in all experiments. Sex-specific outcomes in rodent models of sepsis have been reported, and these differences result from male and female sex steroids performing diverse immunomodulatory functions [17]. Therefore, we used only male mice in this study to obtain consistent data. Animals were housed in a temperature-controlled room with a 12-h light/12-h dark cycle and fed a standard Purina rodent chow diet. Mice were allowed to acclimate to the environment for at least 7 days before being used in experiments. All experiments were performed following the National Institutes of Health guidelines for experimental animals and were approved by the Institutional Animal Care and Use Committee (IACUC) of the Feinstein Institutes for Medical Research. The number of animals in each experiment was determined using SigmaPlot 12.5 (Systat Software, Inc.), and these predictions were in line with our previous publication [18].

Sepsis was induced in mice by cecal ligation and puncture (CLP) [18]. In brief, mice were anesthetized with 2% isoflurane inhalation. The abdomen was shaved and disinfected using povidone-iodine. A 1.5-cm midline incision was made, and the cecum was exposed and ligated with 4-0 silk suture 1 cm proximal from the distal cecal tip. The cecum was punctured twice with a 22-gauge needle, and a small amount of cecal content was extruded. The cecum was then returned to the abdominal cavity, and the wound was closed in layers. Sham mice underwent laparotomy only, without any cecal ligation or puncture. Both sham mice and septic mice received a subcutaneous (*s.c*.) injection of 500 μl of normal saline as resuscitation. Septic mice received an injection of the antibiotic imipenem at a dose of 0.5 mg/kg in 500 μl of normal saline during resuscitation. We also used a single dose (*s.c*., 0.05 mg/kg in 100 µl) of buprenorphine as an analgesic, which was not found to alter results in our previous study, in which we showed that a single dose of buprenorphine given immediately after CLP surgery did not affect serum levels of cytokines [19]. According to the IACUC’s recommendation for animal protocols with Category E pain, we injected septic mice with imipenem (a broad spectrum antibiotic) and an analgesic, buprenorphine, *s.c*. at the end of the CLP operation, following the doses reported in our prior study [19]. Of note, the antibiotic was injected *s.c*. only into septic mice. Sham mice were not injected with imipenem, given that they were normal mice without elevations in inflammatory or injury markers in the serum. Moreover, there was no bacterial load in the blood or peritoneal cavity fluid in normal mice. A previous study clearly indicated that the number of *Candida albicans* ingested by macrophages was not affected by imipenem [20]. In our present study, both WT and CIRP^−/−^ mice were injected (*s.c*.) with the same dose of imipenem immediately after CLP. This experimental approach should have normalized any potential effects of imipenem on bacterial phagocytosis to a comparable level between the WT and CIRP^−/−^ mice. At 72 h after CLP, blood and peritoneal fluid were collected. Mice were anesthetized with 2% isoflurane inhalation to collect blood and peritoneal macrophages. After sample collection, mice were euthanized by CO_2_ asphyxiation, as approved by our IACUC. The same anesthesia and euthanasia procedures were also used in our recent publication [14]. To study survival, mice were monitored twice daily for 10 days, and their survival status was recorded. eCIRP-neutralizing Abs were used in this experiment to inhibit eCIRP after sepsis. The neutralizing anti-CIRP Ab was generated in-house as described previously [11, 21]. One hundred microliters of eCIRP-neutralizing Ab (2 mg/kg BW) or normal saline (vehicle) was administered via retro-orbital injection immediately after CLP. Blood and peritoneal fluid were collected 48 h after CLP to assess the bacterial load.

### Isolation of peritoneal macrophages and cell culture

Male adult mice were anesthetized with 2% isoflurane inhalation. Peritoneal cavity cells were isolated by washing with cold Hanks’ balanced salt solution (HBSS) without Ca_2_^+^ and Mg_2_^+^ with 2% FBS. After peritoneal cell collection, the mice were euthanized using CO_2_ asphyxiation. The collected peritoneal cells were washed once with cold HBSS by centrifugation at 300 × *g* and 4 °C for 10 min, followed by RBC lysis with 0.5 ml of RBC lysis buffer (BD Biosciences) for 5 min at room temperature. F4/80^+^ peritoneal macrophages were isolated using an EasySep mouse F4/80 positive selection kit (Stemcell Technologies, Vancouver, Canada). F4/80^+^ macrophages were cultured in RPMI 1640 medium supplemented with 10% heat-inactivated fetal bovine serum (FBS), 2 mM glutamine, 100 IU/ml penicillin‒streptomycin, and 25 mM HEPES (complete RPMI medium) at 37 °C in 5% CO_2_. Isolated primary peritoneal macrophages were cultured overnight before use. The mouse macrophage cell line RAW 264.7 was obtained from American Type Culture Collection (Manassas, VA) and cultured in Dulbecco’s modified Eagle’s medium (DMEM) containing 10% FBS, 2 mM glutamine, and 100 IU/ml penicillin‒streptomycin (complete DMEM). RAW264.7 cells were cultured at 37 °C in 5% CO_2_.

### Treatment of macrophages with recombinant mouse CIRP and inhibitors

Peritoneal macrophages in complete RPMI medium and RAW 264.7 cells in complete DMEM were cultured overnight, and then the media were changed to Opti-MEM medium 2 h prior to treatment. Recombinant mouse CIRP (rmCIRP) was prepared in-house, and quality control assays were performed as described previously [11]. The quality of the purified protein was assessed by Ponceau staining and Western blotting. A functional assay was performed with the protein by assessing the TNFα levels in macrophages after treatment with purified rmCIRP. The level of LPS in the purified protein was measured by a Limulus amebocyte lysate assay kit (Lonza, Basel, Switzerland). Only the purified protein lots that were free from endotoxin were considered for experiments. We performed these quality control assays for each purified protein lot. rmCIRP at doses of 0.1, 0.2, and 1.0 µg/ml was used in experiments. The STAT3 inhibitor stattic (3 µM) was added 15 min before rmCIRP treatment. The Rac1 inhibitor NSC 23766 (30 µM) was administered 15 min before adding pHrodo-labeled *E. coli* to macrophages for phagocytosis.

### Bacterial culture

At 20, 48, and 72 h after CLP, mice were anesthetized with an overdose of 2% isoflurane inhalation. Blood was collected from the inferior vena cava. Peritoneal lavage fluids were collected after the injection of 1 ml of sterile normal saline into the peritoneal cavity. The blood and peritoneal lavage fluid samples were serially diluted at 1:10, 1:100, and 1:1000 in sterile normal saline, spread on trypticase soy agar plates with 5% sheep blood (catalog no: 221239, BD Biosciences) and cultured at 37 °C for 24 h. Then, the colonies on the plates were counted. We counted the CFU of serially diluted nonconfluent plates with ImageJ software, opting in the threshold, binary, and watershed tools. Others have previously reported the use of ImageJ for counting CFU [22]. Data are expressed as CFU/ml.

### In vitro phagocytosis assay

A total of 1 × 10^5^ peritoneal macrophages or RAW 264.7 cells were seeded in 96-well black tissue culture plates with a transparent bottom (Thermo Scientific) and 100 µl of opsonized pHrodo-labeled *E. coli* (3 × 10^7^ *E. coli*) was added to each well. After incubation at 37 °C for 1.5 h, the phagocytosis efficiency was evaluated by measuring the fluorescence signal using a BioTek Synergy Neo2 multimode reader (BioTek) with an excitation wavelength of 490 nm and an emission wavelength of 533 nm. The cells were then fixed with 4% paraformaldehyde, and the nucleus was stained with DAPI. Fluorescent images of cells were recorded. In an additional group of RAW 264.7 cells, phagocytosis kinetics were measured using a BioTek Cytation image reader (BioTek). Time-lapse images of the phagocytosis of opsonized pHrodo-labeled *E. coli* were taken using a Nikon Eclipse T*i* microscope at 37 °C and a moisture-controlled incubation chamber. RAW 264.7 cells were cultured in plates with a glass coverslip on the bottom (Mat Tek Corporation, Ashland, MA) and pretreated with either PBS or rmCIRP (1 µg/ml) for 20 h; then, pHrodo-labeled *E. coli* (5 × 10^7^/ml) were added to the cells, and time-lapse images were recorded for 1 h at 1-min intervals.

### In vivo phagocytosis assay

rmCIRP at a dose of 5 mg/kg body weight in 400 µl of normal saline or normal saline as the vehicle control was administered into the peritoneal cavity (i.p. injection). The in vivo dose of rmCIRP (5 mg/kg BW) was chosen based on our previous studies, which revealed that at this dose, rmCIRP could induce systemic inflammation and acute lung injury [14, 23]. At 20 h after rmCIRP injection, 3 × 10^8^ pHrodo Green-labeled *E. coli* were administered into the peritoneal cavity by i.p. injection. One hour after the injection, the mice were sacrificed, and peritoneal exudate cells were harvested by collecting the peritoneal lavage fluid through the injection of 8–10 ml of PBS twice. Peritoneal macrophages were then labeled with Pacific blue-conjugated anti-F4/80 antibodies. Macrophage phagocytosis was analyzed using a BD Fortessa flow cytometer. The phagocytosis efficiency was calculated from the median fluorescence intensity (MFI) of pHrodo Green in F4/80^+^ macrophages.

### Confocal microscopy studies

RAW 264.7 cells were seeded in a glass-bottomed chamber slide (catalog no: CCS-8, MatTek Corporation) and fixed with 4% paraformaldehyde for 15 min at room temperature. After washing with PBS, the cells were permeabilized with 0.1% Triton X-100 in PBS for 10 min at room temperature, followed by staining with Alexa Fluor 488-labeled phalloidin (catalog no: 8878, Cell Signaling Technologies) for F-actin and DAPI for nuclear staining. For immunostaining, cells were incubated with anti-Rac1 (Sigma, 1:100) and anti-p-cofilin (Cell Signaling Technology, 1:100) antibodies overnight at 4 °C. After three washes with PBS, Alexa Fluor 568-labeled anti-mouse IgG and Alexa Fluor 594-labeled anti-rabbit IgG were added to the cells and incubated for 1 h at room temperature. The slides were rinsed with PBS three times and mounted with Vectashield plus antifade mounting medium with DAPI (Vector Lab). The slides were examined under a ZEISS LSM 880 confocal microscope (Carl Zeiss Microscopy).

### ELISA

The plasma and peritoneal fluid levels of eCIRP were determined by using an ELISA kit (catalog no: LS-F16777-1) from LifeSpan Biosciences (Seattle, WA). ELISA was performed following the instructions from the manufacturer.

### GTP pull-down assay, immunoprecipitation, and Western blotting

Rac1 activation was determined with a Rac1 activation magnetic bead pull-down assay kit (catalog no: 17-10394, Millipore-Sigma) following the manufacturer’s instructions. RAW 264.7 cells were stimulated with rmCIRP (1 µg/ml). The cells were harvested, and proteins were extracted using the lysis buffer from the assay kit. GTPase proteins in the cell lysate were pulled down by magnetic beads, and the eluate was separated on 4–12% gradient polyacrylamide gels. GTPase-Rac1 was then detected by Western blot analysis. A sample supplemented with a nonhydrolyzable analog of GTP (GTPγS) was used as the positive control, and a sample supplemented with GDP was used as the negative control. For immunoprecipitation, RAW 264.7 cells were treated with rmCIRP (1 µg/ml). The cells were harvested and lysed in lysis buffer (10 mM Tris-HCl at pH 7.5, 100 mM NaCl, 1 mM EDTA, 1 mM EGTA, and 1% Triton X-100) containing a protease inhibitor and phosphatase inhibitor cocktail (Thermo Fisher Scientific). Equal amounts of protein from cell lysates were incubated with a primary antibody against βPIX or STAT3, and the immune complexes were collected with Pierce protein A/G magnetic beads (catalog no: 88804, Thermo Scientific). Following immunoprecipitation, the samples were eluted in sample buffer and run on 4–12% gradient polyacrylamide gels for electrophoresis. Then, the gels were transferred to nitrocellulose membranes. After blocking with 0.1% casein in Tris-buffered saline for 1 h at room temperature, the membranes were incubated with primary antibodies (specific for p-STAT3, STAT3, ARP2, p-cofilin, cofilin, Rac1, βPIX, and β-actin) overnight at 4 °C. The blots were washed with Tris-buffered saline and incubated with secondary antibodies that were labeled with an infrared dye (Li-Cor Biosciences, Lincoln, NE). The protein bands were detected using an Odyssey Clx imaging system from Li-Cor Biosciences and quantified with Image Studio 5.2 software (Li-Cor Biosciences).

### Computational modeling of the βPIX and STAT3 interaction prediction

Computational models can predict the orientation, affinity, and interaction of a ligand in the binding site of a protein. The amino acid sequences of mouse STAT3 (P42227) and βPIX (Q9ES28) were retrieved from the UniProt database. Template-based models were generated using the Iterative Threading ASSEmbly Refinement (I-TASSER) server [24]. The templates were identified by a threading approach to maximize the percentage identity, sequence coverage, and confidence. βPIX has domains including the calponin homology domain (1-112 aa), SH3 domain (163-222 aa), DH domain (250-430 aa), and PH domain (452-557 aa). STAT3 has an SH2 domain (580-670 aa) and a motif (150-162 aa) essential for nuclear transport. The models were refined based on repetitive relaxations by short molecular dynamics simulations for mild (0.6 ps) and aggressive (0.8 ps) relaxations with a 4-fs time step after structure perturbations. Model refinement enhanced certain parameters, including Rama favored residues and a decrease in poor rotamers. Site-specific phosphorylation in the STAT3 structure model was induced using the Vienna PTM 2.0 tool [25]. The docking of the STAT3 and βPIX and p-STAT3 and βPIX protein structure models was performed using the ATTRACT tool [26], which uses an approach of conformational flexibility of binding partners. The docking process includes precalculation of potential energy on a grid, and then interactions are calculated by interpolation from the nearest grid points. Moreover, the docking process includes several Monte Carlo simulations or energy minimization steps. STAT3-βPIX and p-STAT3-βPIX complex interactions were calculated using the PDBePISA tool [27]. The surface area of the interaction interface and thermodynamic parameters were calculated. The complex structure was visualized using PyMOL and Chimera [28].

### Statistical analysis

All data were tested for normality. Parametric data are expressed as the mean ± SD. One-way/two-way ANOVA and the Student-Newman‒Keuls (SNK) test were performed to compare multiple groups. An unpaired two-tailed Student’s *t* test was used for 2-group comparisons. Nonparametric data are expressed as the median ± interquartile range and were compared by the two-tailed Mann‒Whitney test. The Geham–Breslow–Wilcoxon test was used for survival comparison. The specific tests used for each graph are identified in the figure legends. A *p* value <0.05 was considered significant.

## Results

### eCIRP impairs macrophage bacterial phagocytosis in vitro and in vivo

**Fig. 1:**
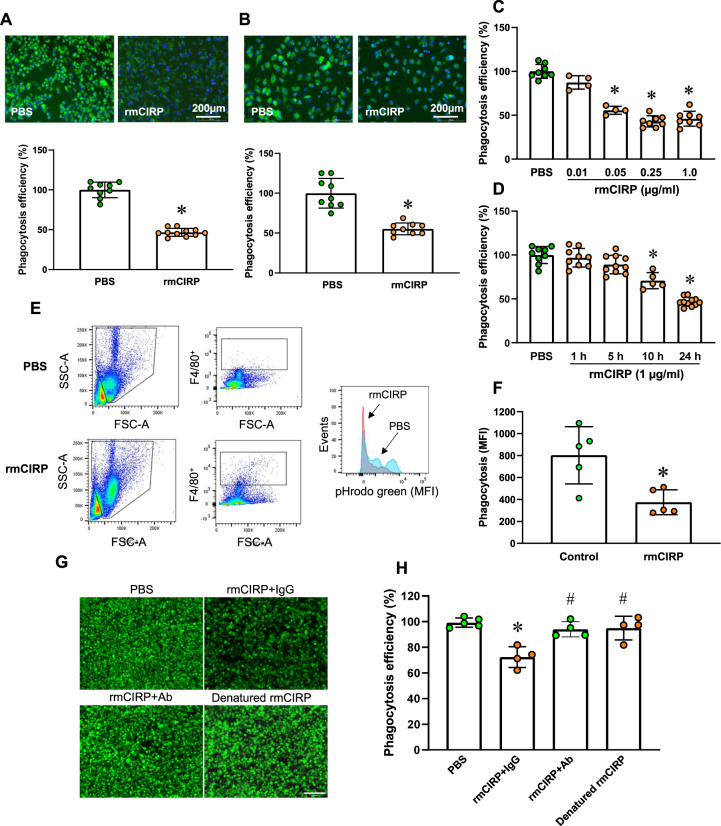
eCIRP impairs macrophage phagocytosis in vitro and in vivo. RAW 264.7 cells (**A**) and murine peritoneal macrophages (**B**) were treated with rmCIRP (1 µg/ml) for 24 h. The cells were incubated with pHrodo green-labeled *E. coli* for 1.5 h. Bacterial phagocytosis was measured using a fluorescence microplate reader. At the end of the phagocytosis period, the cells were fixed, and microscopy images were acquired. Scale bar, 200 µm. The experiment was repeated 3–5 times. The data presented were combined from two independent experiments and are expressed as the mean ± SD (*n* = 9–11/group). The groups were compared by a two-tailed Student’s *t* test. *p < 0.05 vs. PBS control. RAW 264.7 cells were stimulated either with different doses of rmCIRP for 24 h (**C**) or with rmCIRP (1 µg/ml) for different times (**D**). The cells were then incubated with pHrodo green-labeled *E. coli* for 1.5 h. Bacterial phagocytosis was measured using a fluorescence microplate reader. Data are expressed as the mean ± SD (*n* = 4–11/group). The PBS control was set to 100% for normalization. The groups were compared by one-way ANOVA and the SNK method. *p < 0.05 vs. PBS control. **E**, **F** rmCIRP (5 mg/kg body weight) in saline or saline alone (control) was injected i.p. into mice. At 20 h after rmCIRP injection, 1 ml of pHrodo green-labeled *E. coli* (3 × 10^8^) was administered into the peritoneal cavity via i.p. injection, and mice were rested for 1 h to allow phagocytosis. Then, peritoneal lavage cells were extracted, peritoneal macrophages were labeled with a Pacific blue-conjugated anti-F4/80 Ab, and phagocytosis was assessed using flow cytometry. The efficiency of phagocytosis is indicated as the median fluorescence intensity (MFI). **G**, **H** RAW 264.7 cells were treated with rmCIRP (1 µg/ml) in the presence of IgG (10 µg/ml) or anti-CIRP Abs (10 µg/ml) for 24 h. In another group, cells were treated with denatured rmCIRP (1 µg/ml, rmCIRP was boiled for 15 min). The cells were incubated with pHrodo green-labeled *E. coli* for 1.5 h. Bacterial phagocytosis was measured using a fluorescence microplate reader. At the end of the phagocytosis period, the cells were fixed, and microscopy images were acquired. Scale bar, 100 µm. The data presented are expressed as the mean ± SD (*n* = 4–5/group), and the experiment was performed twice. The groups were compared by one-way ANOVA and the SNK method. *p < 0.05 vs. PBS; ^#^*p* < 0.05 vs. rmCIRP + IgG

To investigate the effect of eCIRP on bacterial phagocytosis, we pretreated RAW 264.7 cells with rmCIRP for 24 h and then added pHrodo green-labeled *E. coli* to the cell culture. The cells were incubated at 37 °C for 1.5 h, and the efficiency of phagocytosis was assessed. PBS-treated macrophages showed a strong fluorescence signal from engulfed bacteria (Fig. 1A). In contrast, rmCIRP treatment dramatically reduced macrophage phagocytosis by 53% (Fig. 1A). We also isolated peritoneal macrophages from healthy mice, treated them with rmCIRP for 24 h, and then evaluated bacterial phagocytosis. Similarly, treatment with rmCIRP markedly reduced phagocytosis by peritoneal macrophages by 45% (Fig. 1B). Although we did not use live bacteria to directly assess bacterial killing as demonstrated previously [29], the status of bacterial killing was indirectly confirmed by using pHrodo-labeled *E. coli* Following bacterial engulfment, the phagosome containing the engulfed bacteria fuses with a lysosome to form a phagolysosome and destroy the engulfed bacteria using hydrolytic enzymes activated in the acidic pH environment of the lysosome. The pHrodo system measures phagocytic activity based on the acidification of the particles ingested. The fluorescence intensity of the pHrodo dye dramatically increases in an acidic pH environment. We aimed to confirm the actual engulfment of bacteria and investigate whether the phagosome containing the engulfed bacteria, which were labeled with pHrodo, merged with the lysosomal compartment in macrophages, and our data showed strong pHrodo fluorescence in macrophages, suggesting that the phagosome containing bacteria merged with the lysosome for destruction/killing (Supplementary Fig. 1 and Supplementary Video 1). We then evaluated the dose- and time-dependent effects of rmCIRP on macrophage bacterial phagocytosis. rmCIRP dose-dependently reduced macrophage phagocytosis (Fig. 1C). We observed a slight decrease at 5 h, a significant reduction at 10 h, and a further reduction at 24 h following treatment of RAW 264.7 cells with rmCIRP (Fig. 1D). We next performed an in vivo phagocytosis assay. rmCIRP was injected intraperitoneally into healthy mice. At 20 h after rmCIRP injection, pHrodo green-labeled *E. coli* was administered into the peritoneal cavity, and phagocytosis was allowed to occur for 1 h. Peritoneal lavage cells were extracted, and peritoneal macrophages were identified by Pacific blue-conjugated anti-F4/80 antibodies. Macrophage phagocytosis was assessed using flow cytometric measurement of pHrodo green fluorescence. The MFI was used to evaluate the levels of phagocytosis in peritoneal macrophages. rmCIRP significantly reduced bacterial phagocytosis by 53% vs. control treatment (Fig. 1E, F). Since gram-positive bacteria, i.e., *Staphylococcus aureus*, cause acute peritonitis [30], we assessed the phagocytosis of pHrodo-labeled *Staphylococcus aureus* by RAW 264.7 cells pretreated with or without rmCIRP. We found that treatment with rmCIRP significantly decreased the phagocytosis of gram-positive bacteria by RAW264.7 cells (Supplementary Fig. 2). Thus, eCIRP impaired bacterial phagocytosis in vitro and in vivo. To determine whether blockade of eCIRP with anti-CIRP Ab treatment reduces the effects of eCIRP on bacterial phagocytosis, we treated RAW 264.7 cells with IgG, an anti-CIRP Ab, or denatured rmCIRP and then assessed phagocytosis. We found that treatment of macrophages with the anti-CIRP Ab significantly improved phagocytosis by neutralizing the effects of eCIRP compared to IgG treatment (Fig. 1G, H). Similarly, denatured (inactivated) rmCIRP had no effect that altered normal phagocytosis (Fig. 1G, H). These data indicate that blocking eCIRP with neutralizing Abs reverses phagocytic dysfunction.

### The systemic and peritoneal bacterial loads are reduced in CIRP-deficient mice after sepsis

**Fig. 2:**
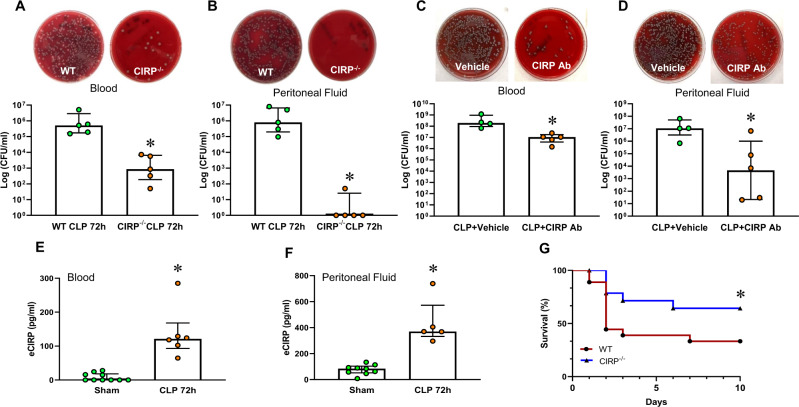
CIRP-knockout mice have a reduced bacterial load in sepsis. **A**, **B** Sepsis was induced in WT and CIRP^−/−^ mice. At 72 h after CLP, **A** blood and **B** peritoneal lavage fluid were collected to evaluate the bacterial load (CFU/ml). Data are expressed as the median ± interquartile range (*n* = 5 mice/group). The groups were compared by the two-tailed Mann‒Whitney test. *p < 0.05 vs. WT. **C**, **D** Sepsis was induced in WT mice with or without anti-CIRP Ab treatment (2 mg/kg BW). At 48 h after CLP, **C** blood and **D** peritoneal lavage fluid were collected to evaluate the bacterial load (CFU/ml). Data are expressed as the median ± interquartile range (*n* = 4–5 mice/group). The groups were compared by the two-tailed Mann‒Whitney test. *p < 0.05 vs. WT. **E**, **F** Sepsis was induced in WT mice. At 72 h after CLP, **E** plasma and **F** peritoneal lavage fluid were collected to evaluate eCIRP levels by ELISA. Data are expressed as the median ± interquartile range (*n* = 5–10 mice/group). The groups were compared by the two-tailed Mann‒Whitney test. *p < 0.05 vs. sham. **G** Sepsis was induced in WT and CIRP^−/−^ mice. The survival curve generated during the 10-day monitoring period is shown. *n* = 18 for WT mice and *n* = 15 for CIRP^−/−^ mice in each group. *p < 0.05 vs. WT, determined by the Geham-Breslow-Wilcoxon test

Delayed pathogen elimination contributes to the detrimental outcomes of sepsis [8]. To investigate the role of eCIRP in bacterial clearance in sepsis, blood and peritoneal fluid were collected at 72 h after CLP. As shown in Fig. 2A, the blood bacterial count was 1.31 × 10^6^/ml in WT mice and reduced to 2.8 × 10^3^/ml in CIRP^−/−^ mice. Similarly, the peritoneal bacterial load was 2.8 × 10^6^/ml in WT mice and dramatically reduced to 10.8/ml in CIRP^−/−^ mice (Fig. 2B). As a therapeutic approach, we treated septic mice with anti-CIRP Abs to target eCIRP. We found that CLP mice treated with anti-CIRP Abs exhibited significantly decreased bacterial counts in the blood and peritoneal fluid compared to vehicle-treated mice at 48 h of sepsis (Fig. 2C, D). Our data showed that the highest levels of eCIRP were found in the blood and peritoneal fluid at 72 h after CLP (Fig. 2E, F and Supplementary Fig. 3A, B), which correlated with the impaired bacterial clearance in these animals. In our in vivo studies with CLP-induced sepsis mice, we collected blood and peritoneal fluid samples to assess the bacterial load at 72 h after the CLP procedure; this time point was chosen since eCIRP levels were highest, which would allow evaluation of the strongest effect on the phagocytic dysfunction of macrophages. Furthermore, we also observed that the 10-day survival rate was markedly higher in CIRP^−/−^ mice (Fig. 2G). Thus, the release of eCIRP in sepsis is associated with an increased bacterial load and septic death.

### eCIRP decreases ARP2 protein expression and cofilin phosphorylation in macrophages

**Fig. 3:**
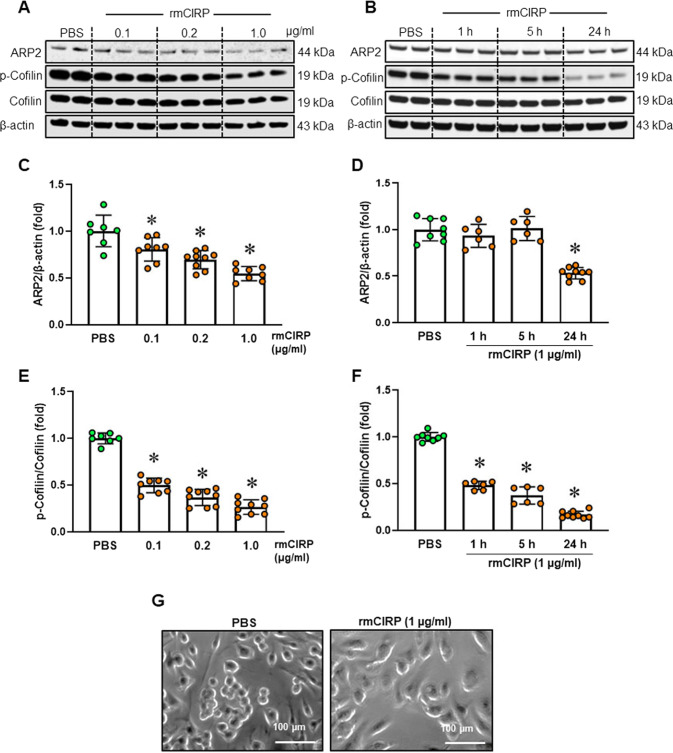
eCIRP downregulates actin-binding protein expression. RAW 264.7 cells were incubated with rmCIRP at doses of 0.1, 0.2, and 1 µg/ml for 24 h (**A**, **C**, **E**) or at a dose of 1 µg/ml for 1, 5 and 24 h (**B**, **D**, **F**). ARP2 and p-cofilin protein expression was assessed by Western blotting. **A**, **B** Representative blots and **C**–**F** the corresponding bar diagram are shown. The experiment was performed three times. The data presented were obtained from three independent experiments and are expressed as the mean ± SD (*n* = 6–9/group). The PBS control was set to 1 for normalization. The groups were compared by one-way ANOVA and the SNK method. *p < 0.05 vs. the PBS-treated group. **G** RAW 264.7 cells were incubated in 1 µg/ml rmCIRP or PBS for 24 h. The cells were examined under a Nikon microscope, and phase-contrast images were acquired. Scale bar, 100 µm

Actin-related protein 2 (ARP2) and cofilin are involved in cell motility [6, 31]. ARP2 mediates actin polymerization, and cofilin disassembles actin filaments to provide G-actin monomers for new filament formation [6]. Our results showed that rmCIRP decreased the ARP2 protein level in a dose- and time-dependent manner (Fig. 3A–D). The expression of ARP2 in macrophages was decreased by 47% at 24 h after 1 µg/ml rmCIRP treatment (Fig. 3D). The activity of cofilin is dependent on its phosphorylation status [31, 32]. Phosphorylation of cofilin was observed in PBS-treated macrophages (Fig. 3A, B, E, F). Cofilin is dephosphorylated in macrophages following stimulation with bacteria [32, 33], but the effect of eCIRP on the cofilin phosphorylation status is unknown. We showed that rmCIRP reduced the phosphorylated cofilin level in a dose- and time-dependent manner (Fig. 3A, B, E, F). A significant reduction in the p-cofilin level was observed even at 0.1 µg/ml rmCIRP (Fig. 3A, E). The p-cofilin level was reduced by 52%, 63%, and 83% at 1, 5 and 24 h, respectively, after rmCIRP (1 µg/ml) treatment (Fig. 3B, F). In addition to ARP2 protein expression and cofilin phosphorylation, we assessed the expression of FcγR1 and found that its expression was not significantly altered in rmCIRP-treated macrophages (Supplementary Fig. 4). The rmCIRP-induced alterations in these actin-binding proteins can affect cell morphology. Macrophages showed enlarged and flattened cell bodies 24 h after incubation with rmCIRP (Fig. 3G). Deficiency in ARP2/3 inhibits lamellipodia protrusion [34], producing a cellular morphology akin to what we observed for eCIRP-treated macrophages (Fig. 3G). Similarly, the phosphorylation of cofilin is an essential regulatory mechanism of actin polymerization and cell membrane protrusion [31]. Even though we did not show an effect of ARP2 and cofilin blockade on cellular morphology, with these supportive prior study findings, our data on the eCIRP-mediated decrease in ARP2 expression and cofilin phosphorylation can be directly correlated with maladaptive membrane protrusions. Thus, eCIRP dysregulates actin-binding proteins and interrupts actin remodeling.

### eCIRP downregulates Rac1 protein expression to decrease bacterial phagocytosis

**Fig. 4:**
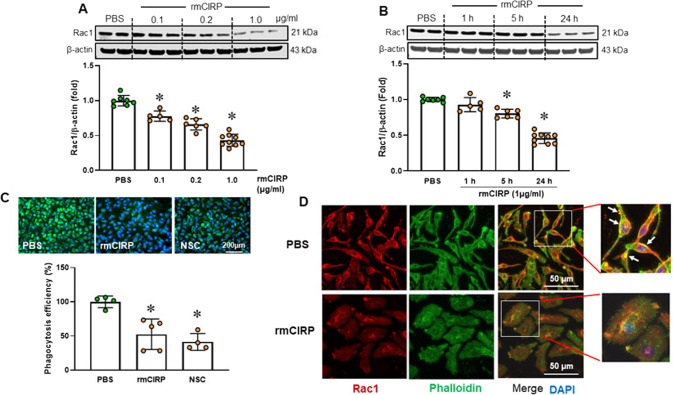
eCIRP downregulates Rac1 protein levels. RAW 264.7 cells were incubated with 0.1, 0.2 and 1 µg/ml rmCIRP for 24 h (**A**) or with 1 µg/ml rmCIRP for 1, 5 and 24 h (**B**). Rac1 protein expression was assessed by Western blotting. Representative blots are shown. Data are expressed as the mean ± SD (*n* = 5–9/group). The PBS control was set to 1 for normalization. The groups were compared by one-way ANOVA and the SNK method. *p < 0.05 vs. PBS. **C** RAW 264.7 cells were incubated with rmCIRP (1 µg/ml) or PBS for 24 h. In an additional group, RAW 264.7 cells were incubated with the Rac1 inhibitor NSC23766 (30 µM) 15 min before adding pHrodo-labeled *E. coli*. The efficiency of phagocytosis was measured using a fluorescence plate reader. The cells were fixed at the end of the phagocytosis assay for microscopy imaging. Representative images are shown. The experiment was performed twice. Data are expressed as the mean ± SD (*n* = 4–5/group). The groups were compared by one-way ANOVA and the SNK method. *p < 0.05 vs. PBS. **D** RAW 264.7 cells were incubated with rmCIRP (1 µg/ml) or PBS for 24 h. Then, the cells were fixed for Rac1 immunofluorescence and F-actin staining. Rac1 (red), F-actin (green), and merged staining images are shown. Framed areas are enlarged to show details. The nucleus was stained with DAPI (blue). Scale bar = 50 µm

We next addressed the mechanism by which eCIRP dysregulates actin-binding proteins. Since ARP2 and cofilin activities are regulated by the signaling GTPase protein Rac1 [6], we investigated the effect of eCIRP on Rac1 expression. rmCIRP inhibited Rac1 protein expression in a dose- and time-dependent manner (Fig. 4A, B). Rac1 expression was reduced by 55% after incubation of macrophages with rmCIRP for 24 h (Fig. 4B). The Rac1 inhibitor NSC23766 suppressed macrophage phagocytosis in a similar fashion as rmCIRP (Fig. 4C), supporting that the inhibition of Rac1 by rmCIRP impaired macrophage phagocytosis. Rac1 immunostaining displayed dispersed cellular staining with a few nuclear localizations in PBS-treated macrophages (Fig. 4D). Rac1 was observed surrounding the plasma membrane in macrophages and colocalized with F-actin (Fig. 4D). Colocalization of Rac1 and F-actin is necessary for Rac1 to regulate actin reorganization in response to stimulation [35]. When RAW 264.7 cells were incubated with rmCIRP for 24 h, the Rac1 signal was markedly reduced, and plasma membrane-localized Rac1 disappeared (Fig. 4D). These data suggest that eCIRP downregulates Rac1 protein expression to decrease bacterial phagocytosis.

### eCIRP decreases the protein expression of βPIX

**Fig. 5:**
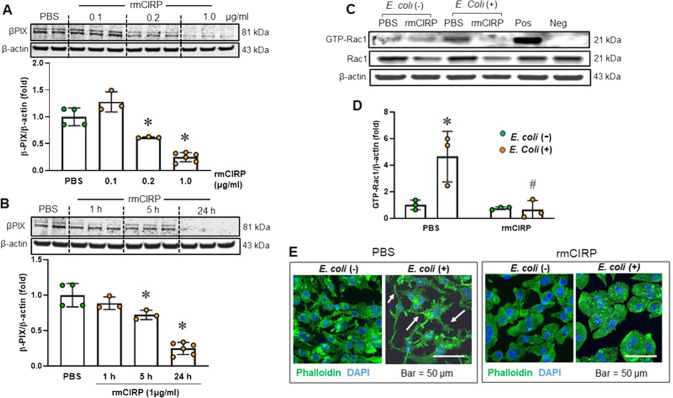
eCIRP downregulates βPIX and inhibits Rac1 activation. RAW 264.7 cells were incubated with 0.1, 0.2, and 1 µg/ml rmCIRP for 24 h (**A**) or with 1 µg/ml rmCIRP for 1, 5 and 24 h (**B**). βPIX levels were assessed by Western blotting. Representative blots are shown. The experiment was performed twice. Data are expressed as the mean ± SD (*n* = 3–6/group). The PBS control was set to 1 for normalization. The groups were compared by one-way ANOVA and the SNK method. *p < 0.05 vs. PBS. **C**, **D** RAW 264.7 cells were incubated with rmCIRP (1 µg/ml) or PBS for 24 h, followed by incubation with or without *E. coli* for 30 min. Then, GTP-Rac1 levels were evaluated by a GTP-Rac1 pull-down assay. The experiment was performed twice. Data are expressed as the mean ± SD (*n* = 3/group). The PBS control was set to 1 for normalization. The groups were compared by two-way ANOVA and the SNK method. *p < 0.05 vs. PBS without *E. coli*; ^#^*p* < 0.05 vs. the PBS-treated group with *E. coli*. Pos positive control, Neg negative control. **E** RAW 264.7 cells were incubated with rmCIRP (1 µg/ml) or PBS for 24 h, followed by incubation with or without *E. coli* for 30 min. Then, F-actin was stained with phalloidin (green), and the nucleus was stained with DAPI (blue). Images were acquired with a confocal microscope, and representative images are presented. Scale bar = 50 µm

We further investigated the upstream regulator of Rac1, βPIX (Arhgef7), a guanine nucleotide exchange factor [36]. βPIX mediates Rac1 activation by promoting the release of GDP from inactive GDP-Rac1 and the binding of GTP to Rac1 (GTP-Rac1) [36]. rmCIRP treatment showed a dose- and time-dependent reduction in βPIX protein expression in macrophages (Fig. 5A, B). βPIX protein expression was reduced by 75% after treatment with rmCIRP for 24 h compared to PBS control treatment (Fig. 5B). We also assessed the mRNA expression of ARP2, cofilin, βPIX, and Rac1 after treatment of RAW 264.7 macrophages with rmCIRP. The results showed that the mRNA expression of ARP2, cofilin, βPIX, and Rac1 was not significantly changed in RAW 264.7 cells following treatment with rmCIRP compared to PBS control treatment, indicating that eCIRP downregulated actin-binding proteins at the posttranscriptional level, independent of transcriptional changes (Supplementary Fig. 5). Furthermore, the downregulation of βPIX by rmCIRP was correlated with the attenuation of Rac1 activation and membrane filopodia formation. Without *E. coli* stimulation, GTP-Rac1 levels were low in both PBS- and rmCIRP-treated macrophages (Fig. 5C, D). After stimulation with *E. coli*, GTP-Rac1 levels were markedly elevated in PBS-treated macrophages to facilitate actin remodeling and phagocytosis. In contrast, GTP-Rac1 levels remained low in rmCIRP-treated macrophages after stimulation with *E. coli* (Fig. 5C, D). In the context of *E. coli* stimulation, PBS-treated macrophages increased membrane pseudopodia, such as filopodia (arrows in Fig. 5E). However, rmCIRP-treated macrophages failed to form membrane projections after *E. coli* stimulation (Fig. 5E). The results indicate that rmCIRP reduces βPIX levels and inhibits the formation of GTP-Rac1. Consequently, actin remodeling is interrupted.

### STAT3 is required for the eCIRP-induced impairment in bacterial phagocytosis

**Fig. 6:**
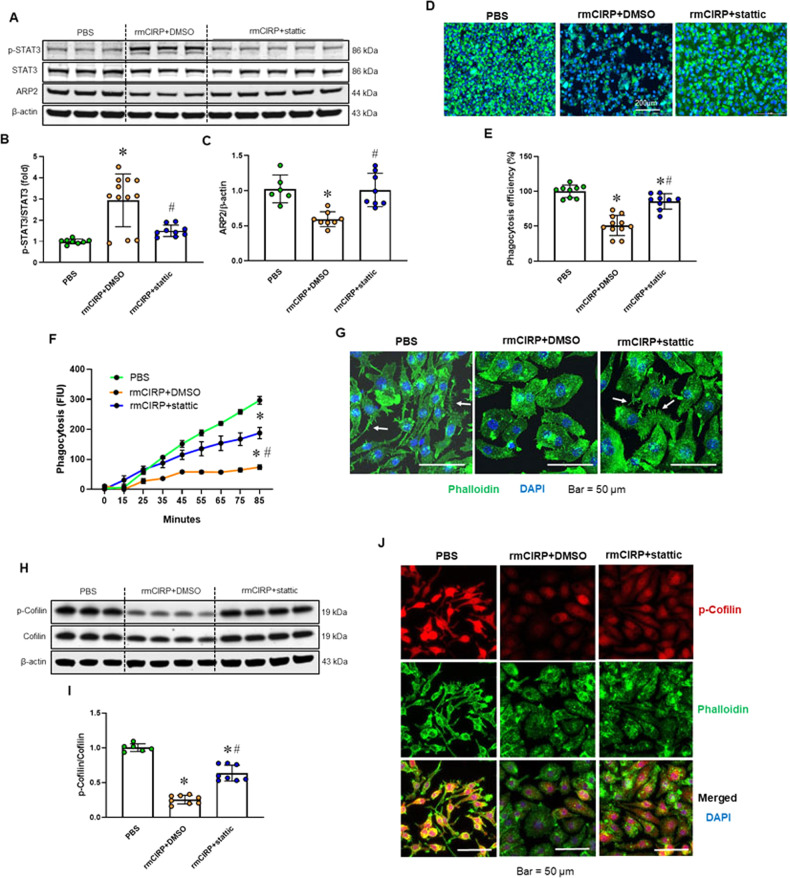
STAT3 activation is crucial to the eCIRP-induced dysregulation of actin remodeling. RAW 264.7 cells were incubated with rmCIRP (1 µg/ml) for 24 h with or without stattic (3 µM). PBS was used as a control. **A**–**C** After treatment, the levels of p-STAT3 and ARP2 were evaluated by Western blotting. Data were obtained from two independent experiments and are expressed as the mean ± SD (*n* = 8–12/group). The PBS control was set to 1 for normalization. The groups were compared by one-way ANOVA and the SNK method. *p < 0.05 vs. PBS; ^#^*p* < 0.05 vs. rmCIRP. **D**, **E** After treatment, phagocytosis was evaluated by adding pHrodo-labeled *E. coli*, and microscopy images were acquired at the end of the assay. Representative images are shown. Data were obtained from two independent experiments and are expressed as the mean ± SD (*n* = 9–11/group). The experiments were repeated five times. The PBS control was set to 100% for normalization. The groups were compared by one-way ANOVA and the SNK method. *p < 0.05 vs. PBS. ^#^*p* < 0.05 vs. rmCIRP. **F** After treatment, the efficiency of phagocytosis was recorded, and the kinetics with 10-min intervals are presented. At the end of the assay, data were analyzed and are expressed as the mean ± SD (*n* = 4/group). The groups were compared by one-way ANOVA and the SNK method. *p < 0.05 vs. PBS. ^#^*p* < 0.05 vs. rmCIR*P*. **G** After treatment, cells were fixed, and F-actin was stained with phalloidin. Representative confocal microscopy images are presented. Scale bar, 50 µm. **H**, **I** After treatment, the levels of p-cofilin were evaluated by Western blotting. Representative blots are shown. The experiments were repeated three times. Data were obtained from two independent experiments and are expressed as the mean ± SD (*n* = 6–8/group). The PBS control was set to 1 for normalization. The groups were compared by one-way ANOVA and the SNK method. *p < 0.05 vs. PBS control; ^#^*p* < 0.05 vs. rmCIRP. **J** P-cofilin immunostaining. P-cofilin (red), F-actin (green), and the merged staining images are shown. Scale bar, 50 µm

STAT3 not only plays critical roles in cytokine signaling and immune responses but also regulates cell mobility in both physiological and pathological conditions [37]. We sought to identify whether the activation of STAT3 is associated with the eCIRP-induced impairment in phagocytic function. As shown in Fig. 6A, B, rmCIRP significantly increased STAT3 phosphorylation, and the STAT3 inhibitor stattic attenuated eCIRP-induced STAT3 phosphorylation. Macrophages treated with stattic alone (3 µM) did not show any noticeable changes in STAT3 activation (Supplementary Fig. 6). Strikingly, inhibition of STAT3 activation restored bacterial phagocytosis in rmCIRP-treated macrophages (Fig. 6D, E). The phagocytosis kinetics curve showed a high upward slope in PBS-treated macrophages over 85 min (Fig. 6F). In contrast, rmCIRP-treated macrophages showed a slow increase in phagocytosis, suggesting that phagocytic function was inhibited in these cells (Fig. 6F). Adding stattic to rmCIRP-treated macrophages markedly increased the slope of the kinetics curve to a level close to that of PBS-treated macrophages (Fig. 6F). Microscopy images indicated that stattic restored the phagocytic function of rmCIRP-treated macrophages. Under bacterial stimulation, stattic increased membrane pseudopodia in rmCIRP-treated macrophages (Fig. 6G). In line with these data, stattic also improved ARP2 and p-cofilin levels in rmCIRP-treated macrophages (Fig. 6A, C, H, I). Macrophages treated with stattic alone (3 µM) did not show any noticeable changes in the levels of ARP2 and p-cofilin (Supplementary Fig. 6). Immunofluorescence staining showed that PBS-treated macrophages demonstrated a strong p-cofilin signal in the cytoplasmic and nuclear compartments (Fig. 6J). P-cofilin levels were dramatically reduced in rmCIRP-treated macrophages, and cytoplasmic p-cofilin was lost. However, stattic significantly restored p-cofilin levels in rmCIRP-treated macrophages (Fig. 6J). Thus, STAT3 activation is critical for eCIRP’s effect on the interruption of actin remodeling.

### eCIRP induces the formation of the STAT3-βPIX complex through the activation of STAT3

**Fig. 7:**
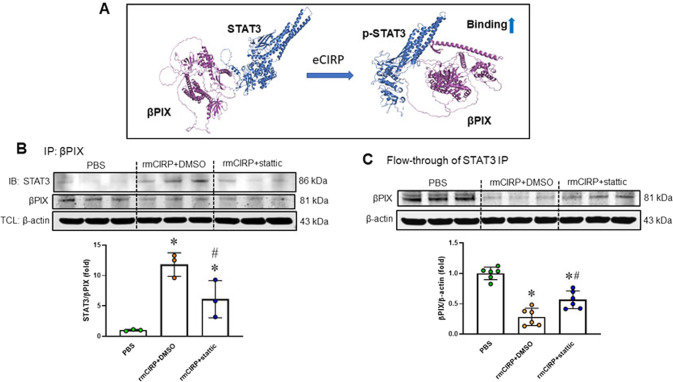
eCIRP induces the formation of the STAT3-βPIX complex. **A** Binding between STAT3 and βPIX was predicted by 3D computational modeling. **B** RAW 264.7 cells were incubated with rmCIRP (1 µg/ml) with or without stattic (3 µM) for 24 h. Then, βPIX was pulled down by immunoprecipitation, and STAT3 levels were determined by Western blotting. Representative blots are shown. The presented data are from one experiment. The experiment was repeated twice. Data are expressed as the mean ± SD (*n* = 3/group). The PBS control was set to 1 for normalization. The groups were compared by one-way ANOVA and the SNK method. *p < 0.05 vs. PBS; ^#^*p* < 0.05 vs. rmCIRP. **C** RAW 264.7 cells were incubated with rmCIRP (1 µg/ml) with or without stattic (3 µM) for 24 h, and then STAT3 was pulled down from the cell lysate by immunoprecipitation. The levels of free βPIX in the flow-through after STAT3 immunoprecipitation were determined by Western blotting. Representative blots are shown. The presented data are from two independent experiments. Data are expressed as the mean ± SD (*n* = 6/group). The PBS control was set to 1 for normalization. The groups were compared by one-way ANOVA and the SNK method. *p < 0.05 vs. PBS control; ^#^*p* < 0.05 vs. rmCIRP. TCL total cell lysate

βPIX is required for Rac1 activation. STAT3 can regulate the activation of Rac1 to mediate cell migration in mouse embryonic fibroblasts through binding to βPIX [37]. We therefore hypothesized that the activation of STAT3 by eCIRP promotes STAT3 binding with βPIX, inhibiting Rac1 activation. We first assessed the interaction between STAT3 and βPIX by computational modeling, which revealed a direct interaction between STAT3 and βPIX (Fig. 7A). Based on the data for the interaction interface surface area in Ȧ^2^, which was 964.6 Ȧ^2^, and other thermodynamic parameters, such as the free energy of binding upon complex formation (^∆i^G) of −4.5 kcal/mol, the STAT3-βPIX interaction was not strong. The free energy of dissociation (∆G^disso^) of the STAT3-βPIX complex was −7.5 kcal/mol. The negative free energy of dissociation indicated that the STAT3-βPIX complex interaction was transient. The entropy change after dissociation (T∆S) was 15.8 kcal/mol. There were 8 hydrogen bonds and 1 salt bridge that contributed to the stability of the STAT3-βPIX complex structure (Table 1). Phosphorylation of STAT3 increased the interaction between STAT3 and βPIX, as indicated by the decrease in the free energy of binding upon complex formation (^∆i^G) to −9.9 kcal/mol and the increase in the free energy of dissociation (∆G^disso^) to −4.7 kcal/mol (Table 1). However, the interaction between phosphorylated STAT3 and βPIX was still an intermediate or transient interaction. The hydrogen bonds between phosphorylated STAT3 and βPIX included Glu510-Lys591 (2.41 Ȧ) and Lys364-Ala598 (2.75 Ȧ). Next, we assessed the interaction of these proteins in cells by immunoprecipitation (IP). We isolated total protein from RAW 264.7 cells treated with rmCIRP or PBS and subjected the total protein to IP with an anti-βPIX Ab. We measured the STAT3 levels in the immunoprecipitant. As shown in Fig. 7B, STAT3 formed a complex with βPIX in rmCIRP-treated macrophages but not in PBS-treated cells. Stattic decreased the formation of the STAT3-βPIX complex in rmCIRP-treated cells. Furthermore, we also performed IP using an anti-STAT3 Ab and then collected the contents with protein A/G magnetic beads. We also collected the flow-through, which included all the proteins except STAT3. We then carried out Western blotting of the flow-through with an anti-βPIX Ab. We found a βPIX band in the flow-through, which did not contain STAT3 or STAT3-bound βPIX, and thus considered this band to represent the free form of βPIX. βPIX levels were significantly reduced in rmCIRP-treated macrophages compared to PBS control-treated macrophages (Fig. 7C). Similarly, inhibition of STAT3 activation by stattic reduced the loss of βPIX through its binding with STAT3 after rmCIRP treatment (Fig. 7C). We noticed that stattic treatment did not completely reverse the effect of eCIRP on the STAT3-βPIX interaction.

**Table 1:** Interaction between STAT3 and βPIX as revealed by computational analysis

Complex	Surface area (Ȧ^2^)	Binding (^Δi^G) energy (Kcal/mol)	Free energy of dissociation (ΔG^diss^) kcal/mol	Entropy change at dissociation (TΔS^diss^) kcal/mol	*N*_HB_	*N*_SB_
STAT3-βPIX	946.6	−4.5	−7.5	15.8	8	1
pSTAT3- βPIX	921.1	−9.9	−4.7	15.4	2	0

This could be because the stattic concentration (3 μM) used in this experiment might have been insufficient to completely reverse the effect of eCIRP on STAT3 related to its binding to βPIX. In addition, the stability of stattic, a chemical compound, might decline with time. Since we treated cells with a single dose of stattic for 24 h, the efficacy in inhibiting eCIRP-mediated STAT3-βPIX complex formation might decrease over time. Administration of a subsequent dose of stattic prolonged the effects attenuating STAT3-βPIX complex formation to a level comparable to that of the baseline control (PBS treatment). Thus, eCIRP-induced activated STAT3 attracts βPIX to form the STAT3-βPIX complex, ultimately reducing the pool of free βPIX. Overall, the binding of STAT3 with βPIX suppresses Rac1 activation, interrupts actin remodeling, and impairs macrophage phagocytosis.

## Discussion

**Fig. 8:**
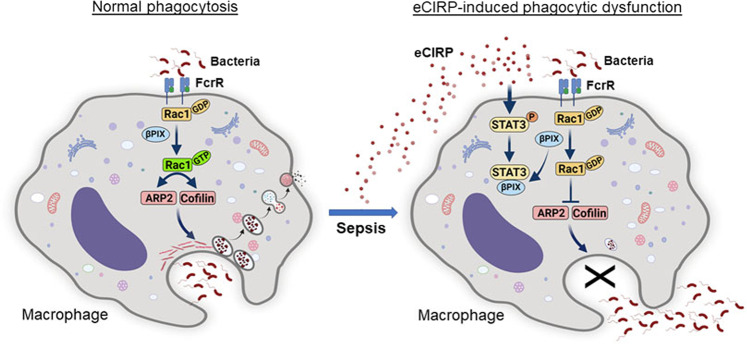
Graphical summary. Under normal conditions, invading pathogens are detected by surface receptors, such as FcγRs on macrophages, and initiate actin remodeling by activating Rac1 to regulate ARP2 and cofilin activities. βPIX regulates Rac1 from the inactive GDP-bound state (GDP-Rac1) to the activated GTP-bound state (GTP-Rac1). Sepsis causes increased release of eCIRP. eCIRP-induced STAT3 phosphorylation causes STAT3 and βPIX to form a complex. Thus, Rac1 activation is inhibited, and actin remodeling is interrupted, resulting in an impairment in the phagocytosis of bacteria

In the current study, we discovered a novel role for eCIRP in inhibiting macrophage phagocytosis of bacteria in polymicrobial sepsis. eCIRP levels were markedly elevated in the plasma and peritoneal fluid after sepsis. A more profound increase in the eCIRP level was found at later time points of sepsis, during which the immunosuppressive phase had started. The increased levels of eCIRP were correlated with a decrease in bacterial clearance in septic animals. Indeed, we found that the bacterial counts in the blood and peritoneal fluid were decreased in CIRP^−/−^ mice with sepsis compared to WT mice. eCIRP inhibited Rac1 activation, causing impaired cytoskeletal rearrangements through eCIRP-induced STAT3-βPIX complex formation. The binding of βPIX with STAT3 led to Rac1 inactivation, inhibiting downstream actin remodeling. Therefore, this study identifies a novel mechanism of macrophage phagocytic dysfunction caused by eCIRP, which may provide a new therapeutic target in sepsis (Fig. 8).

Phagocytic receptors detect opsonized or nonopsonized pathogens. Nonopsonic receptors on phagocytes directly recognize the molecular patterns present on the particle to be engulfed [6]. Opsonic receptors detect host-derived proteins, such as IgG antibodies and complement factors bound to foreign particles [6]. We also revealed that the efficiency of bacterial engulfment was significantly higher for opsonized bacteria than for nonopsonized bacteria (data not shown). Therefore, we used IgG-opsonized bacteria throughout the study. Fcγ receptors (FcγRs) on macrophages recognize IgG-opsonized molecules [6, 38]. Our data showed that the expression of FcγR1 was not altered in rmCIRP-treated macrophages, highlighting eCIRP’s impact on intracellular signaling that affects actin modeling, cytoskeletal rearrangements, and, finally, the engulfment of bacteria. The dynamic expression of the actin-binding proteins ARP2/3 and cofilin is highly relevant to actin remodeling [6, 34, 39]. The generation of actin filaments maintains membrane protrusions (i.e., lamellipodia and filopodia) associated with cell motility and phagocytosis [9, 40]. We revealed that eCIRP downregulated ARP2 expression in macrophages, which ultimately led to decreased numbers of membrane projections, affecting phagocytosis. In addition to ARP2, cytoplasmic cofilin plays a pivotal role in regulating actin cytoskeletal rearrangement [31]. Our data showing decreased cytoplasmic p-cofilin levels in rmCIRP-treated macrophages further support eCIRP’s effects that impair actin remodeling. ARP2 and p-cofilin are downstream molecules of the Rac-1 signaling pathway that directly control cytoskeletal rearrangements. The activation of Rac-1, in turn, requires coupling with βPIX. We found that eCIRP treatment of macrophages significantly decreased the protein levels of βPIX in a dose- and time-dependent manner. However, we did not find any noticeable difference in the mRNA level of βPIX expression after eCIRP treatment of macrophages, indicating that the decrease in the βPIX protein level could be due to the interaction of βPIX with other molecules. Since we determined that eCIRP treatment of macrophages increased the interaction between STAT3 and βPIX, this could cause reduced levels of free βPIX for the interaction producing Rac-1 activation and subsequent induction of the Rac-1-mediated expression of ARP2 and p-cofilin. Therefore, the decrease in the βPIX level through the interaction of βPIX with STAT3 might play an important role in the downregulation of ARP2 and p-cofilin levels. Similar to eCIRP’s effects, HMGB1 deficiency restoring Rac1 activation was previously reported [41], but the impact on Rac1’s immediate downstream molecules was not identified. Our findings of the eCIRP-mediated decreases in ARP2 expression and cofilin phosphorylation address the downstream fates of Rac1 by DAMPs.

Rac1 is a small signaling G protein belonging to the Rho family of GTPases [9] that serves as a master regulator of actin cytoskeletal rearrangement, filopodia formation, and bacterial phagocytosis [33]. In the presence of eCIRP, Rac1 expression and activation were markedly decreased. Colocalization of Rac1 and F-actin is important for Rac1’s regulation of actin remodeling [35]. Rac1 and F-actin were colocalized and surrounded the plasma membrane in untreated macrophages. Such colocalization was not observed in rmCIRP-treated macrophages, indicating the loss of Rac1 regulation of actin remodeling. Coincidently, rmCIRP-treated macrophages formed fewer membrane protrusions when encountering bacteria. βPIX regulates Rac1 from an inactive GDP-bound state (GDP-Rac1) to an activated GTP-bound state (GTP-Rac1) [36]. Rac1 and βPIX interactions are required for Rac1 activation [36]. We found that eCIRP downregulated βPIX levels. In addition, βPIX and STAT3 formed a complex following eCIRP treatment. Thus, eCIRP blocked βPIX from interacting with Rac1, subsequently inhibiting Rac1 function. We further discovered that STAT3 phosphorylation was necessary for the formation of the STAT3 and βPIX complex. Inhibition of STAT3 phosphorylation by stattic reduced the levels of the eCIRP-induced STAT3-βPIX complex, restored the levels of ARP2 and p-cofilin, and, more importantly, recovered phagocytic function. Our findings included both up- and downstream pathways of Rac1 involved in actin cytoskeletal remodeling to establish eCIRP’s novel effects on bacterial phagocytosis. We found that treatment of RAW 264.7 cells with rmCIRP significantly decreased the levels of βPIX in whole-cell lysates. We did not evaluate whether the downregulation of βPIX expression was due to its interaction with activated STAT3, which binds βPIX. Although the mRNA expression of βPIX was not changed in eCIRP-treated macrophages, due to the formation of the STAT3-βPIX complex, the amount of free βPIX was reduced in whole-cell lysates, which could explain the reduced βPIX level in the whole-cell lysates. Similar to our findings, other groups have reported decreased expression of βPIX in aged tissues and cells in mice [42]. Teng et al. reported that STAT3 bound to the monomeric form of βPIX, affecting βPIX oligomerization and resulting in the suppression of βPIX-induced Rac1 activation [37]. This means that the decreased levels of βPIX due to increased STAT3-βPIX formation affect βPIX oligomerization and supports our notion of how eCIRP treatment reduces βPIX levels in macrophages, affecting Rac1 activation and subsequently impairing bacterial phagocytosis.

STAT3 is a critical regulator of cell migration. Teng et al. [37] demonstrated that STAT3 deficiency in mouse embryonic fibroblasts led to elevated Rac1 activity, which enabled a random mode of migration in the cells by reducing directional persistence and the formation of actin stress fibers. They demonstrated that STAT3 negatively regulated the activation of Rac1 to mediate persistent directional migration. They also showed that STAT3’s interaction with βPIX regulated Rac1 activity to modulate the organization of the actin cytoskeleton and directional migration. In contrast to that study on embryonic fibroblasts, our study dealt with terminally differentiated immune cells that serve as professional phagocytes, namely, macrophages. Due to differences in cell type, the intracellular signal transduction leading to the functional outcomes could be different. They compared the impact of the STAT3–βPIX–Rac1 pathway on differential cell migration, i.e., random vs. directional migration. The loss of STAT3 might impair directional migration but promote random or spontaneous migration through βPIX-mediated Rac1 activation. As such, the overall migratory ability might not be impaired, even though the mode of migration was changed from directional to random. Chemotaxis and phagocytosis are two different physiological events in cells. Chemotaxis causes immune cells to migrate at the site of infection, while phagocytosis eliminates bacteria. Although our focus was not to assess the effects of eCIRP on macrophage chemotaxis, given that STAT3 regulates directional chemotaxis, which may cause increased cell migration, the enhanced chemotaxis of migrated cells may not be helpful for the clearance of bacteria given the observed impairment in phagocytosis.

STAT3 is activated through several cytokines, including IL-6 and IL-10 [43], and growth factors, including epidermal growth factor [44] and fibroblast growth factor [45]. Therefore, eCIRP-mediated STAT3 activation could be promoted through these cytokine or growth factor signaling pathways. Indeed, our previous study revealed that eCIRP interacted with the IL-6 receptor (IL-6R), through which STAT3 was activated, and that blocking IL-6R using neutralizing Abs decreased STAT3 activation in macrophages [15]. However, treatment with IL-6R-neutralizing Abs did not rescue eCIRP-induced bacterial phagocytic dysfunction in macrophages (data not shown). This finding indicates the involvement of other receptor(s) in eCIRP-induced STAT3 activation or the possibility that blocking IL-6R may promote different rescue pathways to counteract the phagocytic dysfunction induced by eCIRP. In fact, like other DAMPs, which have binding affinity for multiple receptors, eCIRP interacts with TLR4 and TREM-1 to activate downstream signaling [11, 14]. Although it is challenging to identify a new receptor for eCIRP other than IL-6R, which is involved in STAT3 upregulation followed by phagocytic dysfunction, using CIRP^-/-^ macrophages/mice, we showed recovery of phagocytic function, rigorously confirming eCIRP’s impact on phagocytic malfunction. Future studies that reveal the pathway involved in eCIRP-induced STAT3 activation for phagocytic malfunction would be of interest.

The use of higher in vitro and in vivo concentrations of rmCIRP than the measured levels of eCIRP in the blood and peritoneal fluid of septic mice was justified by the following points. First, it is well known that inflammatory mediators, such as cytokines and DAMPs, work in different modes under in vitro vs. in vivo conditions. In general, much higher doses are required to generate comparable biological effects in in vitro than in vivo, given that in vivo systems include complex cellular crosstalk. Second, RAW 264.7 cells were stimulated with various doses of rmCIRP for 24 h. It was highly possible that the half-life of rmCIRP was significantly reduced over time due to the release of proteinases by RAW 264.7 cells. As such, an increased in vitro dose of rmCIRP was needed to achieve the optimum effect of rmCIRP on bacterial phagocytosis. Indeed, we revealed that >60% of exogenous rmCIRP was degraded within 5 h, while at 24 h of incubation, exogenous rmCIRP was entirely degraded (data not shown). Third, the rmCIRP used in this study (as in all our previous studies) was a His-tagged protein [11]. It is possible that the His-tag modification may somewhat reduce the activities of rmCIRP. Fourth, in contrast to the finding that the rmCIRP degradation rate is higher in vitro, in sepsis, eCIRP is continuously released from activated cells, which maintains adequate eCIRP levels in the blood and peritoneal fluid to execute its harmful effects. This may partially explain the apparently low levels of eCIRP in septic animals. Fifth, again regarding the relatively low levels of eCIRP in the blood and peritoneal fluid under septic conditions, it is highly possible that a significant portion of eCIRP may be bound to its cell-surface receptors (i.e., TLR4, TREM-1, and IL-6R), as these receptors are highly expressed in immune and other cells during sepsis. We previously determined that eCIRP showed strong binding affinity for TLR4, TREM-1, and IL-6R at *K*_D_ values of 6.17 × 10^−7^ M, 11.7 × 10^−8^ M, and 9.8 × 10^−8^ M, respectively [11, 14, 15]. Therefore, the cell-surface levels of eCIRP under in vivo conditions may be much higher than the fluid levels.

Several studies previously investigated targeting eCIRP using knockout mice and peptide antagonists to ameliorate sepsis, hemorrhagic shock, and gut, liver, and renal ischemia‒reperfusion (I/R) injuries [10]. In these studies, the primary focus was on eCIRP’s inflammatory effects mediated through chemokine/cytokine release, inflammatory cell migration, barrier dysfunction, and controlling eCIRP-induced inflammation to obtain favorable outcomes. However, our current findings identify a new pathophysiology of eCIRP that causes impaired bacterial phagocytosis by activating STAT3, sequestering βPIX, and downregulating Rac1 and its downstream molecules ARP2 and p-cofilin, ultimately leading to impaired actin cytoskeletal rearrangement. In addition to polymicrobial sepsis, postsurgical complications and sterile inflammation caused by gut, liver, or renal I/R injury lead to remote organ injury due to gut bacterial translocation or nosocomial infection [46, 47] Moreover, elevated serum eCIRP levels were detected not only in sepsis patients and experimental animals but also in several inflammatory disease conditions. The increased bacterial load and high levels of eCIRP in these inflammatory diseases can be directly correlated and may serve as prognostic markers. Our findings on eCIRP’s effects on bacterial clearance in sepsis can be replicated in other sterile and nonsterile inflammatory diseases to determine novel pathophysiological and therapeutic avenues targeting eCIRP.

In summary, eCIRP dysregulates key signaling pathways involved in actin cytoskeleton reorganization during phagocytosis. The release of eCIRP in sepsis is associated with an increased bacterial burden and mortality. Targeting eCIRP may ameliorate sepsis-induced immunosuppression by restoring typical host defense against infectious pathogens.

## Supplementary information

Supplemental Figures

Supplemental Video 1
