# Supplementary material for: Extracellular CIRP dysregulates macrophage bacterial phagocytosis in sepsis
Source: Cell Mol Immunol. 2022 Dec 5;20(1):80–93. doi: 10.1038/s41423-022-00961-3 (PMC9794804; doi:10.1038/s41423-022-00961-3)
Supplement: Supplementary file 1 — Supplemental Figures [file 41423_2022_961_MOESM1_ESM.docx]

**Supplemental Figures**

**Supplemental Figure 1.** **The effect of eCIRP on macrophage bacterial phagocytosis.** RAW264.7 cells were treated with rmCIRP (1 μg/ml) for 24 h. Cell membranes were labeled with Membrite (green fluorescence) 20 min before adding pHrodo labeled bacteria (Red fluorescence). The cells were incubated in a temperature- and moisture-controlled chamber for phagocytosis (pHrodo labeled *E. coli* 4 × 10^7^/ml). The time-lapse images are recorded every 10 min for 60 min. Cell membrane: Green; *E. coli*: Red; Scale bar = 10 µm.

**Supplemental Figure 2.** **eCIRP impairs macrophage phagocytosis of Staphylococcus aureus, a Gram-positive bacterium *in vitro*.** RAW264.7 cells were treated with rmCIRP (1 μg/ml) for 24 h. The cells were incubated with pHrodo green conjugated S. aureus for 1.5 h. Bacterial phagocytosis was measured using a fluorescent microplate reader. At the end of the phagocytosis, cells were fixed to take microscopic images. Scale bar, 100 μm. The experiment was performed 2 times. Data presented were combined from two independent experiments and expressed as mean ± SD (n = 5/group). The groups were compared by a Two-tailed Student's t-test. **P* < 0.05 versus PBS control.

**Supplemental Figure 3.** **Circulating levels of eCIRP in sepsis. (A)** Sepsis was induced in adult male WT mice. At 20 h and 72 h after CLP, plasma was collected to evaluate eCIRP levels by ELISA. Data are tested for normality of distribution and expressed as median ± interquartile range (n = 6-10 mice/group). The groups were compared by Kruskal-Wallis and Dunn’s multiple comparison method. **p* < 0.05 versus sham. **(B)** Sepsis was induced in adult male CIRP^-/-^ mice. At 72 h after CLP, plasma was collected to evaluate eCIRP levels by ELISA. Data are expressed as mean ± SD (n = 4-5 mice/group). No statistically significant difference between the groups was found.

**Supplemental Figure 4.** **The effect of rmCIRP on FcγR expression in macrophages**. RAW264.7 cells were incubated with rmCIRP 1 µg/ml for 1 h, 5 h and 24 h. FcγR levels were assessed by Western blotting. Representative blots are shown.

**Supplemental Figure 5.** **The effect of eCIRP on ARP2, cofilin, βPIX, and Rac1 mRNA expression.** RAW264.7 cells were treated with rmCIRP (1 μg/ml) for 24 h. RNA was extracted to measure mRNA expression by reverse transcription and real-time PCR analysis. Mouse β-actin mRNA levels were used for normalization. Relative mRNA expression was quantified by the 2^−ΔΔCt^ method. Data were expressed as mean ± SD (n = 4-8/group). mRNA levels in the PBS group are designated as 1 for comparison. The groups were compared by a Two-tailed *Student's t*-test. No statistical difference was found between the groups. Sequences of Primers are below:

| **Name** | **Accession#** | **Forward sequence** | **Reverse sequence** |
| --- | --- | --- | --- |
| ARP2 | BC027799.2 | CACAATCCAGGCAGCCGACATT | AGCTCTCGTTCCAACCTCGATG |
| Cofilin | D00472.1 | GATGCTGCCAGACAAGGACT | GCTCTTGAGGGGTGCATTCT |
| βPIX | NM_001113518.2 | TGGTTGCAGTGTCTATGGGT | GTCTTCTGGGTCAGCAGAGT |
| Rac1 | NM_001347530 | GGGACACAGCTGGACAAGAA | AGATCAAGCTTCGTCCCCAC |
| β-actin | NM_ 007393 | CGTGAAAAGATGACCCAGATCA | TGGTACGACCAGAGGCATACAG |

**Supplemental Figure 6.** **The expression of p-STAT3, ARP2 and p-cofilin at protein levels in macrophages treated with or without stattic.** RAW264.7 cells were incubated with PBS ± stattic (3 µM) for 24 h. Cells were lysed, and protein was extracted. pSTAT3, ARP2 and p-cofilin levels were assessed by Western blotting. Data are expressed as mean ± SD (n = 4/group). PBS only treated group was normalized as one. No statistically significant difference between the groups was found. Representative blots are shown.

**Supplemental Video 1**

Time-lapse images of the phagocytosis of opsonized pHrodo E. coli. were taken using a Nikon Eclipse T*i* microscope under a temperature and moisture-controlled chamber. RAW 264.7 cells were pretreated with either PBS or rmCIRP (1 µg/ml) for 20 h, then pHrodo labeled E. coli. (5 × 10^7^/ml) were added into the cells, and time-lapse images were recorded for 1 h at 1 min intervals.
